# Gender analysis of the World Health Organization online learning program on Immunization Agenda 2030

**DOI:** 10.3389/fgwh.2023.1230109

**Published:** 2023-12-12

**Authors:** Boetumelo Julianne Nyasulu, Shirin Heidari, Michela Manna, Jhilmil Bahl, Tracey Goodman

**Affiliations:** Department of Immunization, Vaccines & Biologicals (IVB), EPI Team, World Health Organization, Geneva, Switzerland

**Keywords:** immunization, child immunization, gender, health, vaccines, agenda 2030, gender-related barriers

## Abstract

Vaccine-preventable diseases pose a significant threat to children under five globally, creating disparities in immunization coverage. Despite its cost-effectiveness and life-saving potential, immunization faces challenges in achieving equitable coverage. Gender inequalities deeply influence access to healthcare, affecting immunization rates. This study examines the action plans submitted by participants of the World Health Organization's (WHO) IA2030 Scholar Level 1 certification course in 2021. A qualitative analysis was conducted on a subset of 111 action plans that scored above 75%, employing narrative thematic analysis to categorize and explore gender incorporation and identified barriers based on the IA2030 Gender Guide. Among the 111 analyzed action plans, gender considerations were present in almost all plans, underscoring the effectiveness of integrating gender perspectives in the course curriculum. The most frequently cited barriers included low education and health literacy, issues related to accessing quality immunization services, gendered dynamics in decision-making within households, and limited access to resources and mobility, predominantly impacting women. The findings confirm that gender inequalities significantly contribute to suboptimal immunization coverage. An intersectional approach, recognizing diverse social markers impacting immunization, is vital to address disparities effectively. Moreover, the need for gender-sensitive data and deeper understanding of intersectional dynamics was emphasized. The study highlights the importance of gender-transformative interventions, including community engagement and efforts targeting both men and women to enhance immunization coverage. While acknowledging limitations, such as potential biases in peer evaluations and the need for wider inclusivity in gender perspectives, this analysis underscores the significance of mainstreaming gender in immunization capacity-building programs. The integration of gender considerations not only raises awareness but also equips professionals to create more gender-responsive immunization programs. Continuous efforts to incorporate gender perspectives can lead to more effective, equitable, and gender-transformative immunization initiatives at various levels.

## Introduction

Vaccine-preventable diseases are a leading cause of death and disability in children under five globally. Vaccine interventions for these diseases are challenged by inequalities in immunization coverage (1). Immunization is a cost-effective health intervention and a key tool in disease prevention. Childhood immunization has significantly contributed to the 59% decline in under-five mortality rates since 1990 (1). The return on investment of immunization interventions is high, with every dollar invested in immunization interventions yielding an estimated direct return of US$20 and an even broader return of US$52 when considering the ripple effect societally (2).

Although there have been notable gains in improving childhood immunization worldwide, immunization coverage remains uneven. In some settings, there is a sizable population with suboptimal immunization and even “zero-dose” children (who have not received any routine vaccination), delaying progress toward eradication of infectious diseases for which effective preventive vaccines exist (3). In 2021, 25 million children missed out on one or more doses of diphtheria, tetanus, and pertussis (DTP) vaccines through routine immunization services, with 6 million more than in 2019, resulting in the highest number since 2009 (4). Of the 25 million, 18 million did not receive a single dose of DTP during the year.

One of the important determinants of under-immunization is gender inequality, which impacts the access to healthcare services, the quality of those services, and vulnerability to disease^1^ (5). Although generally, immunization coverage of boys and girls appears to be equal, gender inequalities and complex gender dynamics influence access to and uptake of immunization services (5). Gender further intersects with other socioeconomic inequalities to impact immunization coverage and health outcomes (5).

Johns et al. (1) conducted a cross-sectional analysis of DTP3 coverage and zero-dose DTP (not receiving any routine DTP vaccination) prevalence in 52 countries using the Survey-based Women's emPowERment Global Index and showed an association between childhood immunization and greater household and decision-making power among mothers (1). Looking at DTP3 coverage as a proxy, they found that the children of women with lower social independence scores were twice as likely to be zero-dose children than those of women with higher social independence scores (1).

In addition, the WHO Strategic Advisory Group of Experts (SAGE) on Immunization in 2007 requested a more detailed analysis of children not reached by immunization. This resulted in investigations on the prevalence of non-vaccination and determinants of zero-dose children. The investigation looked at 241 nationally representative household surveys in 96 low- and middle-income countries and concluded that the main barriers to immunization are lower wealth, the low education status of the caregiver, the low education status of the partner of the caregiver, and the type of family member involved in treatment-seeking decisions (6).

Furthermore, a systematic review of 25 qualitative studies by Merten et al. (6) examining gender-related barriers to childhood vaccination in low- and middle-income countries identified three broad themes. The first theme covered barriers to accessing vaccinations that included gender aspects such as cost and resource allocation, particularly for single women and poor families; lower decision-making power for women; and maternal, domestic, and social tasks that fall on women and create time constraints for health-seeking behavior. An additional barrier identified under this theme was constrained mobility due to social norms and poverty and shame felt by women avoiding scrutiny of the healthcare system. The second broad theme was knowledge and its effect on demand, which included education, health literacy, experiential knowledge, and non-Western medical beliefs in communities that create barriers to immunization. Finally, the last theme was trust in services (distrust in health systems, politically motivated rumors, and population development policies). The systematic review found that resistance to vaccination was higher in countries that underwent coercive population control by governments in the past—when governments of developed countries had economic aid, support, or compliance dependent on developing the acceptance of countries regarding contraception or vaccinations. In addition, the authors found that women's agency was constrained by social and cultural norms, limited resources, and limited decision-making power. On top of that, child health services create a dilemma as they often hold a bias toward women being the sole caregivers while simultaneously compounding blame for those unable to overcome structural or social constraints to immunization-seeking behavior (6).

The Gender and Immunization Summary Report (2010) produced by the WHO Initiative for Vaccine Research adopted a framework for a gender analysis of barriers to immunization and predictors of unvaccinated children based on a statistical analysis of 166 demographic health surveys in 67 countries, a qualitative systematic review and case studies. The findings of this report indicated that there is a gendered aspect of childcare and health that assumes the health status of a child as the primary responsibility of mothers. While this is perpetuated in both traditional and biomedical health systems, mothers are faced with resource and decision-making constraints as often fathers or extended family members hold decision-making power and control of resources (7) because prevalent gender stereotyping perpetuates the notion of mothers as primary caregivers (8). In addition, immunization services rarely target men (7). In many countries, men have power, control over, and access to resources, finance, information, and transportation—which are all key elements for accessing immunization services (7, 8). Consequently, immunization programs often overlook the role of men in decision-making and their participation in immunization services. These findings highlight how harmful gender norms, gender-based violence, unequal access to resources, unequal distribution of power and decision-making, and limited mobility of women shape obstacles to full immunization coverage.

**Figure 1:**
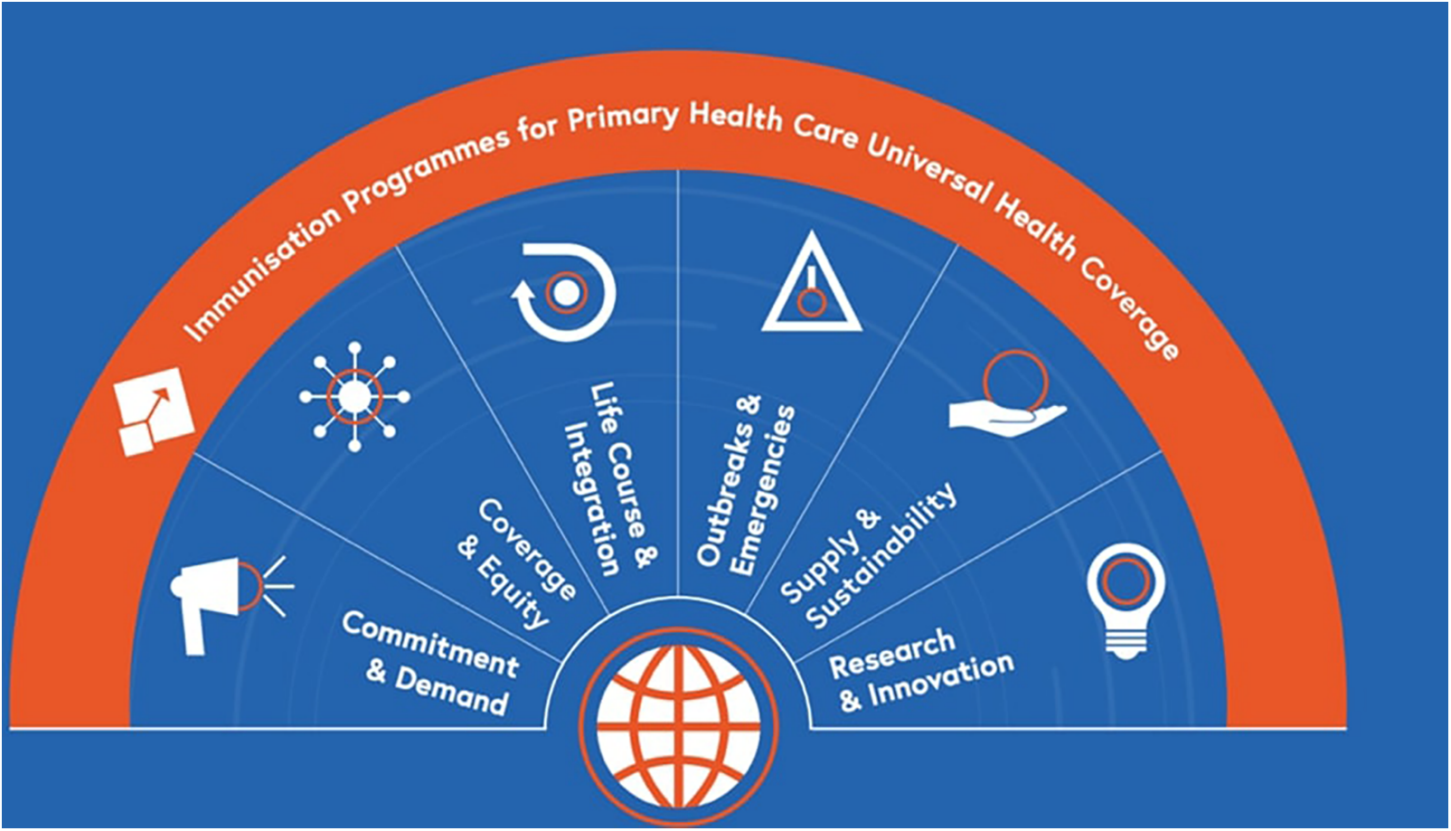
The immunization agenda's seven strategic priorities (9).

The Immunization Agenda 2030 (IA2030) (9) is a global strategy released in 2020 by the World Health Assembly, with the support of countries and partners, to maximize the lifesaving potentials of vaccines and leave no one behind in immunization efforts. The IA2030 recognizes gender as an important cross-cutting dimension for all its seven strategic priorities (see Figure 1 for the seven priorities) and commits to addressing gender-related barriers to immunization and advancing gender equality to realize its vision. To this end, accompanying the IA2030, the immunization partners published “Why Gender Matters: Immunization Agenda 2030” (10) (hereafter referred to as the IA2030 Gender Guide). The IA2030 Gender Guide aims to improve the understanding of the gender-related barriers to immunization and offers recommendations, practical tools, and effective actions to mainstream gender into immunization programs as a way to improve immunization coverage.

To accelerate awareness about the IA2030 and its accompanying Gender Guide and to strengthen capacity, the WHO offers a 6-week online certification course (WHO IA2030 Scholar Level 1 course) that invites applicants involved in national or sub-national immunization planning. Upon completion, the participants are expected to be familiar with the strategic priorities and core principles of IA2030 and be equipped to plan and implement the activities that maximize the impact of immunization efforts. During the course, the participants are asked to prepare an action plan that:

1. identifies the strategic priorities and focus areas that are likely to transform their national immunization program and the work of global partners
2. defines the problem and prioritizes recommendations for a suggested action that could have the greatest impact on the current state of their country
3. identifies the best practices for the suggested action through literature review and dialogue with their peers
4. explores the innovative ways to implement strategic priorities in their country context by applying the knowledge and skills learned during the course

The peer-to-peer learning of the program allows the participants to review and grade the action plans of their peers based on a standard rubric to provide a peer review score. To strengthen the gender competencies and knowledge of gender-related barriers in immunization, in line with its commitments toward gender mainstreaming, a gender module was introduced in the WHO Immunization Scholar Programme in 2020. In 2021, in addition to the dedicated module on gender and immunization, gender was mainstreamed throughout the course curricula, and the participants were asked to identify gender-related barriers and integrate gender dimensions into their action plans to improve immunization coverage.

The action plans that were submitted by the participants for the IA2030 Scholar course in 2021 were analyzed to assess the extent of successful gender mainstreaming in the action plans. The analysis further identified the common gender-related barriers reported by the course participants and the approaches proposed to address and overcome these barriers.

## Methodology

The action plans from the first cohort of 2021 were used for this analysis and reviewed and coded by one researcher. In total, 256 action plans were submitted by course participants. A quantitative analysis of the action plans was conducted using Microsoft Excel to categorize the gender of the participant, the country of focus of participants in the action plans (see Table 1), the average peer rating of the action plan, the health system level (see Table 2), and the frequency at which specific gender-related barriers were cited.

**Table 1:** Gender of course participants and countries of focus in action plans (*N *= 111).

Country of focus (*N *= 111)	Man	Woman
Bangladesh	1	0
Burkina Faso	2	1
Colombia		1
Democratic Republic of Congo	1	1
Egypt		1
Ethiopia	2	2
Gambia	2	2
Ghana	8	3
Guinea	1	
India	8	2
Indonesia	1	1
Jamaica		1
Jordan	1	
Kenya	1	5
Liberia	3	
Malawi	1	
Nigeria	11	19
Pakistan	4	3
Panama	1	
Papua New Guinea	1	
Philippines		2
Sierra Leone		1
South Korea	1	1
South Sudan	1	1
Sri Lanka		1
Tanzania		1
Timor Leste		1
Turkey		1
Uganda	5	2
Yemen		1
Zimbabwe	1	
Total	57	54

**Table 2:** Health system level of action plan sample (*N *= 111).

Health system level	Number of action plans in the sample
District (or equivalent)	17
Global	3
Health facility (or equivalent)	12
Multicountry	3
National	39
Other sub-national (province, state, zone or region, etc.)	37
Total	111

A sample of action plans with a peer review score above 75% (111 out of 256) were selected to carry out a qualitative analysis. A narrative thematic analysis of the action plans was used to examine the incorporation of gender and categorize the identified barriers. A narrative thematic analysis is a qualitative research approach that identifies the recurring themes and patterns in the narrative data, such as interviews or written accounts, to understand the underlying meanings and experiences of the subjects (11). It helps researchers uncover and interpret the key themes within the narratives for a deeper understanding of the data. The analytical categories used for the narrative analysis were based on the five main gender-related barriers to immunization outlined in the IA2030 Gender Guide. While the IA2030 Gender Guide resource does not stand alone as a gender analysis framework, it offers a relevant structure for organizing information about gender barriers to immunization given the focus of the IA2030 course.

The barriers above were used to analyze each action plan; the sub-codes that are included in Table 3 summarize what emerged from the narrative analysis using an inductive method of coding where sub-codes are derived from the data directly. After collating the data into the code sets marked by the five gender-related barriers, each code set was reviewed to assess the proposed approach to addressing the barrier. Gender-responsive approaches to overcoming the barriers were organized using the IA2030 Gender Guide categories (see Table 4).

**Table 3:** Immunization Agenda 2030 (IA2030) Gender Guide gender-related barriers and the emergent sub-codes.

Barrier to	Sub-codes
Quality services and health provider attitudes	- Factors related to the quality, accessibility, and acceptability of immunization services, such as opening hours, distance, waiting times, hygienic standards, and provider attitude - The quantity, gender balance, attitude, and behavior of healthcare workers and other gender barriers health providers themselves face
Education and health literacy	- Factors related to literacy level, access to health information, knowledge of utilization, and access to immunization services - Educational level and health literacy of parents and caregivers
Decision-making and household dynamics	- Factors related to social gender roles in the household that influence the control over their individual and children's health decisions - Gender power relations within the household that designate caregiver and decision-making roles and the level of bargaining power in the household
Access and control over resources and mobility	- Level of access and control of resources including time (particularly influenced by the burden of responsibility of household work, caretaking, and family obligations), money and information, and gender differences in mobility due to transportation costs, availability, and the safety and security of certain areas especially in conflict or emergency settings
High prevalence of gender-based violence and harmful practices	- Experiences of gender-based violence and its consequences on physical, psychological, sexual, and reproductive health and its effect on health-seeking behavior and use of reproductive, maternal, and child health services - The impact of child marriage on the access of girls and women to and utilization of health services, particularly immunization, through mobility restrictions, impact on education levels and bargaining power on health decisions, and son preference impact on the immunization uptake of girls

**Table 4:** IA2030 Gender Guide gender-responsive approaches with descriptions.

IA2030 Gender Guide: gender-responsive approach	Description
Invest in gender data and analysis	Disaggregated data are systematically broken down by sex, gender, age, location, disability, ethnicity, socioeconomic status, and others followed by analysis, collection, monitoring, and evaluation
Make community engagement and social mobilization gender-responsive and transformative	Social mobilization and community engagement of groups and the design of immunization materials that challenge gender norms and communication channels and platforms that understand and cater to the different needs of men and women to improve health education/literacy
Engage with men to transform gender norms	Inclusion of men in outreach and messaging, communications, engagement in groups or associations, and training of health personnel
Empower and collaborate with civil society and change agents	Partnerships with civil society and change agents including grassroots groups, informal community networks, men, women, and youth groups, marginalized groups, or gatekeepers in different contexts to inform the design and delivery of services and increase immunization demand
Implement gender-responsive actions for the health workforce	Safeguarding mechanisms for gender-based violence, gender equality training for health workers, and addressing gender inequalities in the health workforce
Improve the quality, accessibility, and availability of services	Addressing locations and environments of health centers and facilities, scheduling and availability of services, and accessibility of these facilities for all
Integrate services and collaborate across sectors	Bundling immunization with other health and non-health services and interventions to improve immunization
Implement gender-responsive immunization services in emergency settings	Immunization services that understand and address different needs, priorities, and roles of people in emergency settings. Particularly, the impact on access to education, health services, timely services, and accurate information
Apply a gender lens to research and innovation	Incorporating the voices of men and women in vaccine research and development, taking into consideration sex and gender when testing to influence gender-responsive implementation research, learning, and innovation

Coding was conducted manually, by rereading the action plan sections and identifying recurring words, prominent ideas, or patterns generated from the data that fell under factors described in the sub-codes. Once narrative blocks were coded and grouped into the five gender-related barriers with the corresponding gender-responsive approaches to those barriers, all the narratives were read over with similarities and differences noted to inform the findings. The last stage of the thematic analysis was interpreting the data to inform an overall gender analysis. After fully exploring the narrative blocks, a core narrative was written, including direct quotes from the action plans, to showcase the main points and commonalities raised under each barrier.

## Findings

### Quantitative analysis

The 256 action plans were submitted by 137 (54%) men and 119 (46%) women. The countries with the highest number of action plans were Nigeria (79), India (24), Ghana (18), Pakistan (14), Kenya (14), Uganda (9), and Ethiopia (9).

For the sample of 111 action plans that had peer scores exceeding 75%, the country representation of each barrier was quite diverse although the high counts of Nigeria, India, and Ghana reflect that these were the countries with the highest number of course participants and action plans submitted (see Table 4 for full breakdown). Similarly, the gender balance of the course participants who submitted action plans in the sample was 57 men and 54 women, with a similar balance of gender representation among the main barriers cited (more information in Table 5). Topics and themes that were more frequently mentioned by women course participants were as follows: the need for male engagement in immunization activities (18/27 of action plans were submitted by women), the health literacy gap caused by information targeting women alone (13/18 plans were submitted by women), and disseminating educational messages to raise community awareness (22/33 plans were submitted by women). Interestingly, the barrier cited by more men than women course participants was the compounded burden of work and caregiving faced by women (5/6 plans by men).

**Table 5:** Barriers identified, gender balance, and country of focus (*N *= 111).

Gender barrier	No. of 111 action plans citing the barrier	Plans submitted by men	Plans submitted by women	Action plans country of focus
Barrier to education and health literacy	58	29	29	Bangladesh, Colombia, Democratic Republic of Congo, Egypt, Ethiopia, Gambia, Ghana, Guinea, India, Indonesia, Jamaica, Kenya, Liberia, Nigeria, Pakistan, Panama, Philippines, South Korea, Timor Leste, Turkey, Uganda, Yemen, Zimbabwe
Barrier to quality services and health provider attitudes	56	29	27	Bangladesh, Burkina Faso, Colombia, Democratic Republic of Congo, Egypt, Ethiopia, Gambia, Ghana, India, Indonesia, Jamaica, Jordan, Kenya, Liberia, Malawi, Nigeria, Pakistan, Papua New Guinea, Philippines, Sierra Leone, South Korea, South Sudan, Uganda
Barrier to decision-making and household dynamics	38	16	22	Burkina Faso, Ethiopia, Gambia, Ghana, Guinea, India, Indonesia, Kenya, Nigeria, Pakistan, Philippines, Sierra Leone, South Sudan, Timor Leste, Uganda
Barrier to access and control over resources and mobility	27	18	9	Burkina Faso, Democratic Republic of Congo, Ethiopia, Ghana, India, Malawi, Nigeria, Pakistan, South Korea, Uganda
Barrier to the high prevalence of gender-based violence and harmful practices	0	N/A	N/A	N/A

Nearly all the action plans included a gender dimension except three that did not explicitly mention a gender-related barrier but incorporated gender consideration in the approach to improve immunization. Nearly half of the action plans [53% (59/111)] included more than one gender-related barrier, resulting in overlapping representation in the data. The only barrier from the IA2030 Gender Guide that was not referred to in any of the 111 action plans was the “prevalence of gender-based violence and harmful practices.” Table 5 provides an overview of the identified barriers.

## Qualitative analysis

### Barrier to education and health literacy

Of the 111 action plans that were examined, the most frequently cited barrier to immunization was low education and health literacy (58/111), highlighting the linkage between the education level of a mother and the immunization status of a child. Of these 58, the majority [(44/58) or 75%] highlight low health literacy and education in communities. Only two of the action plans specifically mentioned lower education and health literacy of men as a barrier and 12 specifically highlighted the low rate of literacy for women. The remaining 14/58 action plans referenced misinformation, immunization information targeting women, and inaccessibility of men to health information. One action plan stated:

Although paternal education is also associated with a child's immunization status, lower educational levels of maternal caregivers are more commonly related to under-vaccination in lower- and middle-income countries. A comprehensive review of immunization equity found that the greatest disparity exists for children with uneducated mothers. A mother's individual educational level as well as the literacy rate of her community are important factors for a child's complete immunization. *(Male course participant, sub-national health system level, SP1, India)*

The action plans shared that the lack of knowledge or incorrect knowledge about immunization services leads to hesitancy or refusal of vaccines. In 15 of 58 action plans citing the barrier to health literacy and education, the participants indicated that male engagement can positively contribute to immunization uptake. Providing health literacy and education at the community level could significantly impact vaccination efforts by tackling misconceptions, beliefs, and distrust of healthcare providers and vaccines.

### Barrier to quality services and health provider attitudes

This second most frequently cited barrier in the action plans covered quality, acceptability, and accessibility of immunization services and was identified in 56 out of 111 action plans. The participants highlighted how the quality, acceptability, and accessibility of immunization services can be influenced by the number of healthcare staff and clinics or hospitals available in a location, the gender of the healthcare staff that caters to the preferences and needs of the community, quality, and hygiene of the environment and respectful attitude of the staff.

The most frequently reported factor [76% (35/56)] by the course participants was the lack of well-trained female healthcare providers or the absence of female healthcare workers altogether. Due to health worker shortages in some areas, outreach services may be scaled back or canceled, and the number of locations where vaccinations could be provided may be reduced, which impacts the access of women to immunization. In addition, in areas with mainly male health workers, where families or women prefer female attendants, this severely impacts immunization uptake. An action plan shared that:

Most health workers (vaccinators) are men and that could limit women's attendance at vaccination sessions, especially in rural areas where women seek care from female providers. *(Male course participant, national health system level, SP3, Burkina Faso)*

The second most cited factor under this barrier [48% (27/56)] called attention to the low engagement of men and fathers at health institutions and the failure to target fathers and men who are care providers and include both genders in health services.

The focus is on maternal and child health, and this is intrinsic and normalized. For instance, HPV vaccines are offered to girls only even though internationally the practice has changed to include males. The culture is that women are responsible for the child's health, and they tend to choose the mother to give the history and at times ask the father to wait outside. *(Female course participant, national health system level, SP4, Jamaica)*

The action plans to highlight gender-related barriers to quality service called attention to an institutional level neglect to ensure gender balance in a workforce that delivers service and care, better engagement of men and fathers at the facility level, and lack of entry points for men. Concerning difficulties in recruiting men, one action plan shared:

There are no male nurses working in mother and child health clinics because male nurses often wish to identify with “masculine” nursing services. *(Female course participant, district health system level, SP7, Kenya)*

This highlights the importance of not placing the blame on healthcare providers, who are often female, for not managing to target or engage men when men may simply not be willing to engage due to the pressure of masculinity. Considering the challenges above, addressing the gender balance of healthcare staff also needs to consider the preferences of communities for certain healthcare activities. This is important not only for encouraging women to attend immunization activities but also for navigating factors that inhibit men from providing or engaging in immunization services.

### Barrier to decision-making and household dynamics

The gender dynamics in household decision-making can also influence access and uptake of immunization services, as identified by 38 out of 111 action plans. The cultural, social, legal, or other restraints can limit the participation of an individual in decision-making, with the majority of action plans [57% (22/38)] under this category addressing the limited autonomy of women. As noted in one plan:

Mothers are considered the primary caregivers for their children but are often not empowered to fulfill this role. Interventions should contribute to learning, empowerment, and forging of links across social groups. *(Male course participant, national health system level, SP3, Uganda)*

More than half of the action plans of this barrier [57% (22/38)—the majority were by women course participants] noted that men make most of the household decisions while they often do not have sufficient information on routine immunization to guide their decisions. Approximately one-third [31% (12/38)] highlighted how women have to “seek permission” from their husbands for their children to be vaccinated in some settings. Four plans, three of which were written by female participants, mentioned the refusal of husbands for reasons such as cost, misconceptions, or simply not seeing the value of immunization coverage.

Men also tend to associate immunization services with maternal health care/women, refusing to take the child themselves, accompany their wives to the health facility, or go into the facility. Most immunization information targets women, further reflecting the stereotype of women as caregivers; while it is important to target women for this reason, it is also important to target men with accurate information about the benefits of immunizations. This gender stereotyping is reproduced in healthcare institutions and makes addressing this barrier imperative not only at the community level but also at the facility level.

### Barrier to access and control over resources and mobility restrictions

Of the 111 action plans, 27 mentioned limited access and control over resources and mobility restrictions as a key barrier to immunization, with 14 of the 27 (51%) stating that women are not allowed to travel to health facilities alone due to gender norms or sociocultural and security reasons. As such, without the support of their husbands, women and their children miss out on vaccination opportunities. More active engagement of men in immunization services, once again, was noted as an important approach to circumvent this barrier. While it would not be gender transformative and does not address the underlying harmful gender norms, it could serve as an interim step to improve immunization.

Of the 27 action plans, 12 (44%) also highlighted that taking children for vaccination often falls under the responsibility of mothers, who also typically carry the burden of household work and family responsibility. Interestingly, 10 of these 12 plans were written by male course participants, identifying that the investment of time to take children for vaccination can often be difficult to commit to as women have to trade family obligations, household work, or income-generating activities for the journey to a health facility.

Travel imposes direct costs associated with transportation and indirect costs associated with wage loss and unpaid care work in the home including childcare. *(Male participant, sub-national health system level, SP1, India)*

Without additional support from their husbands or other family members, the value of child or individual immunization is not weighed as heavily against other family obligations, household work, or income-generating work. Such a concern was not raised for men, once again highlighting how immunization is mainly seen as a responsibility of women even among course participants.

Missed vaccination opportunities can be further exacerbated for children from single-mother households where mothers hold all financial and family responsibilities—as noted in an action plan from Ethiopia:

Gender may affect immunization services through various ways. For example, about 70 percent of immunization service is provided by health posts staffed by female health extension workers. Females are the predominant vaccination service providers in Ethiopia. However, children from female-headed households and single mothers tend to be unvaccinated and drop out from service compared with children having both parents. Children from single-mother households tended to miss out on more vaccination services and opportunities compared to the children from two-parented households. *(Male participant, SP1, Ethiopia)*

Furthermore, approximately half (51%, 14/27) of action plans mentioned how mobility concerns can be aggravated by far distances to health facilities, poor transportation means to facilities, and even weather conditions that make it difficult to travel long distances, for example, during floods or rainy seasons. These are barriers for both men and women. Cultural expectations and other gender norms may limit the independent movement of women in public, which can result in mobility restrictions for women. For disadvantaged populations settled in urban slums or at the periphery of health facilities or services, there are greater access barriers and thereby higher risks of missing vaccinations as health facilities are often further away, and there is a lack of accessible transportation.

Travelling long distances to health clinics may delay women, particularly younger mothers, from bringing children for immunization due to safety and mobility issues. *(Female participant, health facility level, SP1, DRC)*

### Gender-responsive approaches in the 111 sample of action plans

It was evident that many of the well-documented barriers in the IA2030 Gender Guide are indeed experienced by course participants, and they offered various approaches and ideas to address these barriers that are also supported in other studies. The participants recognized that for health systems to be gender-responsive, research must generate gender-sensitive evidence and that data must be collected and reported disaggregated by sex and other necessary inequality dimensions. It was proposed in 38 action plans [34% (38/111)] to mainstream gender into immunization by investing in gender data and analysis.

Developing an easy-to-use data entry and analysis tool can help the EPI team identify coverage gaps by village, sex, and other parameters. The monthly information [can] be evaluated using gender as one parameter to see the coverage among the two genders. Improvement plans [can] be developed after reviewing the gender-based data. *(Female course participant, sub-national health system level, SP7, Timor Leste)*

The specific recommendations were to break down the collection of data systematically by sex and additional factors including age, location, socioeconomic background, disability, and ethnicity to identify intersectional gender inequalities and contribute to the design of appropriate and contextual gender-responsive approaches. In addition, these action plans suggested the inclusion of qualitative methods to understand embedded gender-related barriers for service providers and recipients.

Innovation in healthcare institutions was highlighted in nine action plans [8% (9/111)] where it was suggested to incorporate gender and sex dimensions in the research process to better contextualize healthcare and make digital healthcare technologies more accessible.

Key importance will be equitable health workers distribution after gender analysis with respect to delivering immunization services at primary health care to improve gender equity. The technology will be gender friendly. To effectively overcome gender-based issues, previous immunization data will be analyzed to identify previous gender-based barriers in the delivery and utilization of immunization services in the communities and primary healthcare centers. *(Male course participant, national health system level, SP1, Liberia)*

There were 59 action plans [53% (59/111)] that proposed approaches for social mobilization and community engagement to improve gender-aware health literacy, with some recommending the dissemination of educational messages and tailored communication strategies. Women were emphasized as a target group in all spheres of the community—encouraging education for girls, strengthening their socioeconomic status, and utilizing outreach groups or organizations of women as an important voice in the community.

In this analysis, 38 action plans [34% (38/111)] proposed community engagement by involving reputable community members that ranged from family members to traditional birth attendants, community focal persons, health workers, village health teams, traditional institutions, local councils, support groups, or women leaders in the community.

Village Health Teams and Opinion leaders at a community and health facility level play a fundamental role in providing care in remote communities and helping to drive health-seeking behaviors. *(Male course participant, national health system level, SP3, Uganda)*

The recommendation to improve immunization coverage through targeting information and messaging toward the engagement of men in caregiving and immunization decisions was included in 30 action plans [27% (30/111)]. For this recommendation, action plans included conducting health promotion activities about the importance of involving male partners in vaccination using radio stations, television stations, community information centers, or even information mobile vans to educate communities. Several also suggested designing immunization materials, messages, and interventions to challenge harmful gender norms, roles, or stereotypes. For example, portraying men as equal and active participants in health-seeking activities and/or showing men caring for children in immunization-related messaging and materials. Promoting health education that explains the importance of male involvement in immunization activities. Many emphasized that men also play a vital role in the planning of dialogs, training, sensitization meetings, and micro plans at the community level.

Gender considerations for the health workforce such as recruitment, training, retention, remuneration, security, and promotion were highlighted in 33 action plans [29% (33/111)]. Approaches suggested incentive schemes, on-the-job support or mentoring, and increased training opportunities especially for female health workers be offered. In addition, these action plans emphasized the importance of a gender-balanced workforce and the standards of the health worker environment.

A majority of the action plans [66% (74/111)] directly addressed the barrier to extending and improving immunization services as critical to expanding coverage and equity. In 18 of these action plans [16% (18/111)], the participants emphasized the need to strengthen existing and open new outreach to reproductive, maternal, neonatal, child, and adolescent health service centers to improve the flexibility and quality of immunization services. Action plans shared that utilizing linkages with other health and non-health interventions can improve immunization uptake and expand immunization reach by building on cost-effective service delivery and institutionalizing certain services and spaces as health outposts. The remaining action plans suggested providing immunization day-offs by employers and including local politicians, religious leaders, community group leaders, and parents in scheduling immunization days to also increase health services availability.

Finally, on a policy level, the main drivers of inequity are governance, stakeholder engagement, health reform programs and policies, laws, and regulations that impact immunization (12). These action plans addressed coverage and equity by putting forward approaches to improve or develop health policies focused on the accessibility of immunization services. This would include distance to the services, transportation, positive inclusion of both genders, and providing income-generating activities to improve the economic status of both men and women for easy access and affordability of services.

Currently, the health policy is focusing on having no woman to carry a child more than five kilometers for immunization services, however, to make it more accessible it will be important to work towards having it at a radius of three kilometers. *(Male course participant, sub-national health system level, SP1, Gambia)*

The action plans suggested that developing or improving health policies focused on the distance to immunization services, providing stipends for transportation, or improving transportation services could make services more accessible. Immunization outposts for routine and supplementary immunization should be in proximity and cover a greater percentage of the population. The distance of the community covered should be considered as it should be reachable by foot without the need for a transport support system. Creating context-based income-generating activities for women will encourage positive inclusion, and improving the economic status of both mothers and fathers could promote easy access to and affordability of services. Leveraging existing funding options can provide support for pro-gender strategies and interventions. Making vaccines available for young girls and women as part of health insurance packages or routine medical examinations required for entry into higher education or employment could increasingly influence uptake. In addition, conducting a mapping of all disadvantaged families and their locations and building on this knowledge contributes to providing people-centric health services. Adoption of participatory methods of service delivery allows families to feel a part of immunization programs.

## Discussion

Our analysis included quantitative and qualitative analysis of 111 action plans that were submitted by the course participants in 2021, who are key actors in planning and delivering immunization services across 31 countries. The findings show that all action plans in the 111 sample, except three, included gender considerations, indicating that mainstreaming gender in the course curricula was an effective strategy to raise awareness among the course participants and draw their attention to gender-related barriers to immunizations. It further encouraged course participants to identify solutions to overcome these gender-related obstacles, as was included in all 111 action plans.

The most cited barrier in the action plans was low education and health literacy as a huge reason for zero-dose or undervaccination. Other barriers most frequently discussed were difficulties in accessing immunization services due to gender-related factors influencing mobility, location, availability, or quality of health services. The lack of involvement of men in immunization decision-making and the impact of men as main household decision-makers on health-seeking behavior were also frequently cited across action plans. The quality and gender balance of health workers, vaccinators, and providers were also highlighted in several action plans.

The proposed strategies of action plans in implementing incentive schemes, on-the-job support or mentoring, and augmenting training opportunities, particularly for female health workers, hold promise for enhancing healthcare services. However, it is imperative to acknowledge and address potential limitations associated with these approaches. While incentive schemes might boost motivation, their long-term sustainability may be uncertain, potentially leading to dependency on external rewards. Ethical concerns may arise due to the creation of disparities in compensation among health workers. Resource constraints could challenge the feasibility of sustaining these measures in resource-limited settings. In addition, a singular focus on incentives might divert attention from essential aspects of healthcare delivery. On-the-job support and mentoring, although valuable, can hinge on the availability of effective mentors and supportive supervisors. Dependency on specific individuals could disrupt the continuity of this support. Moreover, approaches that target only female health workers for increased training might inadvertently reinforce gender stereotypes or overlook broader systemic issues. These limitations underscore the necessity of a comprehensive and contextually sensitive implementation strategy that carefully considers potential challenges to ensure the success and sustainability of the proposed interventions and holds the potential for transforming harmful gender norms.

Drawing from the experience of course participants who have expertise in immunization programs across 31 countries, our findings confirm that gender inequality and harmful gender norms in many settings create barriers and are the main reasons for suboptimal immunization coverage. Indeed, many of the gender-related barriers outlined in the IA2030 Gender Guide are experienced by course participants, who also offered various approaches and ideas to address these barriers.

Our findings also highlight areas where certain gender norms persist. Among others, the widely held assumption that mothers as the ones responsible for health services and immunization was also common among course participants (13). Child health services often target women, considering them as the sole caregivers, while neglecting to address the limited resources and access of women, which may result in compounding blame on women who are unable to overcome their structural constraints for child vaccination (6). While women are often delegated the responsibility of the health of their child, they have to negotiate that with lower educational and social status, less income, and limited decision-making power (6).

Feletto and Sharkey (12) suggested that the key to improving immunization programs and reducing gender inequity within immunization rests on understanding the socioecological contexts in which immunization takes place. This requires a two-pronged approach of first recognizing the heterogeneity of women and the complex intersecting dimensions and experiences of exclusion of different groups and populations of women (12). This requires an intersectional perspective that acknowledges how gender interacts with other social markers such as socioeconomic status, education, age, class, caste, religion, and others to compound disparities within immunization.

As indicated in the action plan findings, there is a high need for sex-disaggregated data and gender-sensitive indicators. There are large evidence gaps that present a barrier to identifying gender-related challenges and potential gender disparities (5). The action plans suggested conducting qualitative research to understand persistent gender-related barriers such as education level, literacy status, employment, purchasing power, and any other sociodemographic characteristics. Data disaggregation by these elements can support in better understanding of the reasons for the low uptake of immunization by parents and caregivers (14). Showcasing the intersection of gender with other social determinants of health can significantly improve health policy and health institutions by making them more gender-sensitive and ideally gender-transformative (5). By embracing the tenets of intersectionality in the realm of immunization activities, a profound shift toward inclusivity and equity can be fostered. The multifaceted nature of the lives of individuals is best understood when gender is examined through its intersection with other factors including but not limited to ethnicity, age, socioeconomic status, and geographical location (15, 16). This approach is necessary to acknowledge the synergistic impact of these intersecting identities and is essential for breaking down systemic barriers. As we delve into this paradigm, we move closer to a reality where immunization activities are not only gender-sensitive but also deeply attuned to the intricate interplay of factors that influence health outcomes.

Behavioral interventions in health need to take intersectional gender dynamics into account. The action plans in this analysis proposed community engagement as a gender-responsive approach to tackle barriers and constraints created by cultural, traditional, and biomedical norms. Similarly, in the evidence review by Kraft et al. (17), they proposed that gender transformative interventions must target adolescent men and women, adult women and men, couples, and the broader community. The proposed approaches focus on empowering women and girls in communication and decision-making by tackling literacy, education, livelihood, and decision-making either at a group or community level.

Additional gender transformative interventions identified at the community level by Kraft et al. (17) were those that focused on the responsibility and joint decision-making of men in the household. Interestingly, many action plans also proposed improving the engagement of men as a possible approach to improving immunization coverage.

In addition, as proposed by Feletto and Sharkey, it is a prerequisite to recognizing the multilevel socioecological aspect of immunization programs that involve the individual, the community, the institution/system, and policies (12). One such approach is to increase the knowledge and awareness of those involved in the planning and implementation of immunization programs about the impact of gender norms, roles, and relations on immunization outcomes.

Following gender mainstreaming in the course curriculum of the WHO IA2030 Scholar program, our analysis confirms the positive impact of this approach. By drawing attention to the importance of gender in immunizations and offering tools on how to identify and address gender-related barriers, the course participants were able to identify gender-related barriers and propose solutions to overcome them.

While our analysis is the first to showcase the positive inclusion of mainstreaming gender in a WHO capacity-building program, it has some limitations. First, past programs of this kind have not been subjected to analysis to evaluate the extent to which gender considerations were integrated into the action plans. However, one of the coauthors, who is the course leader of the program and familiar with the content and action plans submitted by the previous course participants, verifies that gender was rarely a topic that was paid attention to in previous courses.

Another limitation is the fact that our qualitative analysis included only a subset of the submitted action plans based on scores following evaluation by peers. The course participants were of varying genders, nationalities, and backgrounds and may or may not have been aware of their own implicit biases they brought into the peer evaluation, which may have impacted the score given. In addition, the level of experience and expertise on gender issues may vary greatly and thus influence the extent to which the participants received or provided peer feedback to strengthen the gender dimensions throughout the course.

Despite these limitations, this analysis supports the importance of mainstreaming gender in immunization capacity-building efforts to raise greater awareness and equip immunization professionals to deliver more gender-responsive immunization programs. This, in turn, has the potential to improve the effectiveness of immunization programs and reach more children and people with vaccines, while at the same time promoting gender equality. Continuous efforts and support to mainstream gender in such capacity-building initiatives are valuable and could contribute to more gender-responsive and even gender-transformative programs and better policy and immunization services at local, sub-national, and national levels.

## Data Availability

The raw data supporting the conclusions of this article will be made available by the authors, without undue reservation.

## Footnotes

^1^
Note: in this manuscript, the following terms are defined by the World Health Organization as follows ***Sex****: The classification of living things, generally as male or female, based on their reproductive anatomy and functions. It is often categorized based on physical characteristics such as genitalia, chromosomes, and hormone levels*. ***Gender****: This refers to the roles, behaviors, activities, expectations, and societal norms that societies consider appropriate for men and women. Gender is not solely determined by biological or physical attributes but is also influenced by cultural, social, psychological, and emotional factors*.

## Author’s note

BN and MM are Consultants to the WHO. SH is an Independent Gender Consultant to the WHO.
