# Corrigendum: Gender analysis of the World Health Organization's online learning programme on Immunization Agenda 2030

**DOI:** 10.3389/fgwh.2024.1433748

**Published:** 2024-06-28

**Authors:** Boetumelo Julianne Nyasulu, Shirin Heidari, Michela Manna, Jhilmil Bahl, Tracey Goodman

**Affiliations:** Department of Immunization, Vaccines & Biologicals (IVB), EPI Team, World Health Organization, Geneva, Switzerland

**Keywords:** immunization, child immunization, gender, health, vaccines, agenda 2030, gender-related barriers

A Corrigendum on Gender analysis of the World Health Organization’s online learning programme on immunization agenda 2030 By Nyasulu BJ, Heidari S, Manna M, Bahl J and Goodman T. (2023) Front. Glob. Womens Health 4:1230109. doi: 10.3389/fgwh.2023.1230109

**Omitted funding and acknowledgments statement**

In the published article, there was an error, resulting in omitting the Funding and Acknowledgments statement. The correct Funding and Acknowledgments statement appears below.

